# Characterization and Performance of a Thermal Camera Communication System

**DOI:** 10.3390/s20113288

**Published:** 2020-06-09

**Authors:** Victor Guerra, Jaime R. Ticay-Rivas, Victor Alonso-Eugenio, Rafael Perez-Jimenez

**Affiliations:** Institute for Technological Development and Innovation in Communications, Universidad de Las Palmas de Gran Canaria, 35017 Las Palmas de Gran Canaria, Spain; jrticay@idetic.eu (J.R.T.-R.); valonso@idetic.eu (V.A.-E.); rperez@idetic.eu (R.P.-J.)

**Keywords:** optical wireless communication, thermographic cameras, peltier cells, channel characterization, terahertz communication, thermal camera communication

## Abstract

This work presents a novel communications technology named Thermal Camera Communication (TCC), which is analogous to Optical Camera Communication (OCC). Thermographic cameras and Peltier cells are proposed as receiver and transmitter, respectively, changing completely their usual field of application. Furthermore, a comprehensive characterization of the Peltier–Thermal camera pair is carried out, presenting their bandwidth, achievable data rate under On-Off-Keying (OOK) modulation, noise characteristics, and energy efficiency. A comparison against the current state-of-the-art OCC technology is also provided, showing that TCC is a promising technology suitable for sensor networks. The thorough analysis of TCC performed in this work shows that commercial Peltier cells can be re-thought under a communications viewpoint in order to improve their performance. This novel communication technology can be applied in environments such as the access to public transportation or buildings due to the new health emergency situation. The use of thermographic cameras will become massive and dual measurement and communication purposes could be considered for applications such as sensor networks, using a yet unexploited wavelength range.

## 1. Introduction

The new global context derived from 2020’s health emergency will extend the use of thermographic cameras to a wide range of novel applications. Temperature monitoring of individuals in airship access control in airports or even in shopping centers or public buildings will become common. The expected massive adoption of thermal cameras will drop their currently elevated cost. This work explores the possibility of carrying out a dual use of these devices, not only as traditional temperature sensors, but also as communication endpoints. This would allow their application, for instance, in thermal-wavelength sensor networks in any scenario in which thermographic cameras were used.

Terahertz spectrum applications such as wireless mobile communications, wireless data centers, and inter/intra-satellite communications [1] are currently being explored. However, there is currently an important limitation regarding the effective power output of THz antennas (usually up to tens of mW) and, therefore, in the achievable link ranges (currently bounded to the centimeter range). Terahertz wave generation is generally a complex procedure and several strategies can be found in the literature. Photoconductive stripline antennas [2,3], quantum cascade lasers [4], and photoionization [5] are the most used techniques. These methods present low conversion efficiencies and in some cases, as quantum cascade lasers, they usually need from cryogenic temperatures.

As it is aforementioned, the controlled generation of THz waveforms is costly and complex. This limitation was smartly overcome by Liang et al. using blackbody radiation [6]. In their communications scheme, a temperature-controlled light source was optically filtered and encoded by occlusion using a mechanical chopper. Data rates up to 640 bps per channel were reported by the authors using a pyroelectric infrared sensor as receiver.

Thermographic imaging is a THz-based technology that has been amply used in applications such as agriculture [7], damage detection in industry [8], and photovoltaic cell inspection [9]. Furthermore, novel biomedical applications of thermal imaging have been reported in the last few years. For instance, Müller et al. demonstrated in [10] the benefits of joining thermal and visible light images in neurosurgery. Despite the wide range of uses of thermal cameras, this type of device has not been considered as a communications endpoint yet (up to the authors’ knowledge). On the other hand, Optical Camera Communication (OCC) has gained momentum in the last few years, and is a technology that promises considerable market adoption in the near future due to the ubiquity and cost-efficiency of embedded optical cameras [11]. Furthermore, thermal imaging has also experienced a notable cost reduction thanks to the use of CMOS readout circuitry and monolithic microbolometers, allowing not only affordable industrial applications of this technology, but also its commercialization as consumer electronics devices. This opens an interesting opportunity for use cases not only in the traditional application domains of this technology, but also in communications.

Resolution is the main inherent limitation of any imaging device with respect to its use as a communications receiver. In OCC, there is a geometrical relationship between emitter size, distance, camera angular resolution, row-scanning time, and the maximum achievable data rate [12]. As the operation principle of thermal cameras is the same as OCC’s, the emitter must present a sufficiently high effective area. This can be obtained either by using big focal lengths, by using expensive high resolution arrays, or by increasing the physical size of the blackbody-based emitter. In this work, Peltier cells are proposed as current-controlled thermal infrared light sources and microbolometer-based cameras as receivers, conforming a new communications paradigm called Thermal Camera Communications (TCC). This communications technology allows a dual and simultaneous use of thermal cameras, since they can still operate for their current purposes whilst data is gathered from a thermal-band sensor network. In this work, the experimental characterization of a Peltier cell–thermal camera pair is carried out, presenting important communications parameters such as bandwidth and energy efficiency. Moreover, a detailed comparison against its visible range analogous technology, OCC, is also presented.

This paper is structured as follows. Section 2 and Section 3 describe the working principle of both Peltier cells and thermographic cameras, respectively. The characterization methodology of the thermal OWC channel used in this work is presented in Section 4 and Section 5 displays the obtained results. A comparison with a similar technology such as OCC is provided in Section 6. Finally, some conclusions are extracted commented in Section 7.

## 2. Peltier Cells

Thermoelectric cooling devices make use of electric energy to generate temperature differences. Thermoelectric phenomena, such as Seebeck effect, Peltier effect, and Thomson effect, occur due to the interaction between electrical currents and heat flow in the cell’s substrate (typically a semiconductor). These interactions allow the usage of thermoelectric effects for cooling (or heating) purposes.

**Figure 1:**
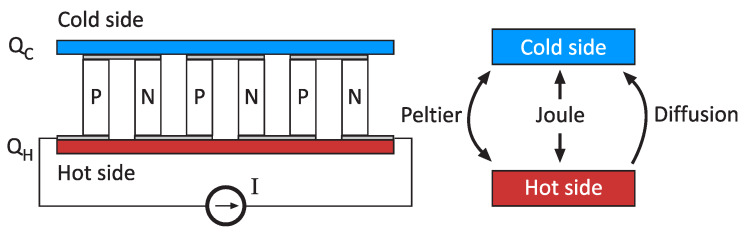
Peltier cell fabrication and heat diagram.

Peltier cells are built using semiconductor substrates such as Bismuth Telluride (Bi${}_{2}$Te${}_{3}$). By defining the appropriate n-type and p-type structure, a temperature difference between two planes can be obtained (Figure 1). Peltier effect forces a temperature difference, which is distorted by the presence of Joule heating due to the driving current. Moreover, there is diffusion between the two plates due to heat transfer.

Peltier cells have been used in many temperature-sensitive scenarios. Najera-Ruiz et al. analyzed the efficiency-enhancement impact of temperature-controlled solar cells using thermoelectric coolers [13], improving the efficiency respect to uncooled operation up to 15%. Peltier cells have been also used for temperature stabilization of wavelength-tunable lasers [14] and high-power light-emitting diode (LED) lamps [15].

Ruiz-Ortega and Olivares-Robles analyzed the transient-state equations (Equation (1)) of a 1D p-type pellet using the Finite Elements Method [16],

(1) $$\frac{\partial^{2}T}{\partial x^{2}}-\frac{I\varepsilon_{t}}{A\kappa }\frac{\partial T}{\partial x}+\frac{I^{2}\varepsilon_{r}}{A^{2}\kappa }=\frac{\rho C_{p}}{\kappa }\frac{\partial T}{\partial t}$$

where *T* is temperature, *x* is the spatial coordinate, *I* is the driving current, $\varepsilon_{t}$ is Thomson effect coefficient (assuming it is constant), *A* is the pellet’s cross section, κ is heat diffusivity, ρ is the material’s density, $C_{p}$ is specific heat, and $\varepsilon_{r}$ is electrical resistivity.

The authors studied supercooling under pulsed-current operation, and found a strong dependence with Thomson effect coefficient and the structure length. From Equation (1) it can be easily observed that the system presents a single-pole response when all the coefficients are assumed constant with temperature (as the authors did). Furthermore, the associated time constant, and therefore the bandwidth, depends on the driving current *I*.

Peltier figure of merit is usually defined as a relationship between Seebeck coefficient (*S*), electrical conductivity ($\varepsilon_{r}^{-1}$), and thermal conductivity (Equation (2)). In practice, there are few materials suitable to be used for thermoelectric applications, and present a figure of merit close to unity.

(2) $$Z=\frac{S^{2}}{\varepsilon_{t}\;\varepsilon_{r}}$$

Recent advances in materials science have shown that the Z≈1 barrier can be raised up [17], opening the possibility to more efficient materials. In this work, thermoelectric coolers are used as wavelength-tunable optical emitters, which is an innovative use of these devices.

## 3. Thermographic Imaging

The aim of thermographic imaging is the bidimensional representation of the thermal infrared (IR) radiation emitted by an object or scene. Thermal IR detectors are classified into photon detectors and thermal detectors. Uncooled thermal detectors are the most used in thermographic cameras due to their small form factor, low power consumption, high cost-efficiency, large spectral response, and long-term operation [18]. Conventional uncooled IR detectors have a small number of sensors (sometimes just only one). These detectors are inadequate in thermal imaging applications since a 2D sensor, also named Focal Plane Array (FPA), is needed for image conformation. There are three main uncooled FPA technologies: microbolometer sensors [19,20], thermopile sensors [21], and pyroelectric sensors [22]. The sensitivity of microbolometer sensors is higher than thermopile sensors. Moreover, their fabrication process is easier than pyroelectric detectors [23].

**Figure 2:**
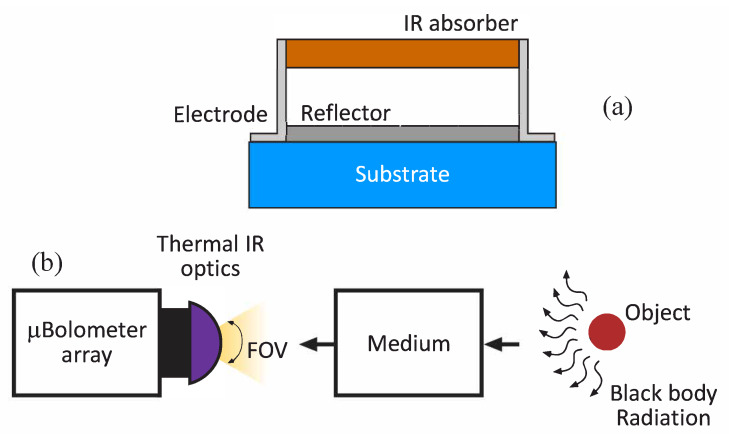
Microbolometer structure (**a**) and thermographic camera scenario (**b**).

Microbolometers are passive devices in which the resistance varies with temperature changes. Those changes are transformed into a measurable quantity (voltage or current). The schematic block diagram of a typical microbolometer detector structure is illustrated in Figure 2a. The incident IR radiation increases the temperature of a material formed on the thermally isolated and suspended bridge, generating a change on its resistance related to its Temperature Coefficient of Resistance (TCR). This resistance change is electrically transferred to the Read-Out Integrated Circuit (ROIC) for further processing. To obtain high sensitivity, the thermometer is kept thermally insulated respect to the ROIC substrate. Figure 2b depicts a generalized use case scenario of thermography. The blackbody radiation emitted by an object propagates through a medium (usually the atmosphere) and is captured by the thermal camera.

The TCR is the first figure of merit that defines the microbolometer performance (Equation (3)) [24],

(3) $$\alpha =\frac{1}{R}\frac{dR}{dT}$$

where α is TCR, *R* is the microbolometer’s resistance, and *T* is temperature. α can be understood as the sensitivity of resistance to temperature. Thus, large α values suggest that a small change in the temperature on the sensing material will result in a large change on its resistance.

The second figure of merit is the voltage responsivity $R_{v}$ (Equation (4)) [25],

(4) $$R_{v}=\frac{I_{b}\alpha R\zeta }{G\sqrt{1+\omega^{2}\tau^{2}}}$$

where $I_{b}$ is the bias current, ζ is the IR absorption coefficient of the material, *G* is the thermal conductance between the detector and the substrate, ω is the angular frequency, and τ is the thermal response time which equals $C_{b}/G$. $C_{b}$ is the specific heat of the microbolometer’s sensitive area. It can be observed that the incident power-resistance response is a low-pass filter. Therefore, the resulting bandwidth limits the maximum frame rate of the thermographic camera.

The third figure of merit is the detectivity ($D^{*}$), which provides the ratio of responsivity to noise per unit bandwidth [26]:

(5) $$D^{*}=\frac{R_{v}\sqrt{A_{eff}\Delta f}}{V_{n}}$$

where Δf is the sensor noise bandwidth, $V_{n}$ is the total root-mean-square sensor noise, and $A_{eff}$ is the effective area.

As it occurs with any imaging device, the projected size of the target object depends on the camera resolution, its Field Of View (FOV), and distance [27]. From a communications viewpoint, due to the a priori slow responses of thermal systems, Rolling Shutter (RS) effect will not be suitable as bandwidth-improving technique in this case, despite the CMOS readout circuitry employed in thermal cameras. Therefore, the encoding techniques must take into account the camera’s frame rate and not the row-scanning time of the sensor.

## 4. Methodology

The main objective of this work is to provide a rigorous characterization of both thermographic cameras and Peltier cells as receivers and transmitters respectively from a communications viewpoint. Concretely, in order to provide useful metrics about the feasibility of the proposed technologies for communication purposes, the system’s current–temperature step response and the noise levels were experimentally obtained. With this information, an estimation of the channel capacity and the energy efficiency (in bits per Joule) can be supplied.

**Figure 3:**
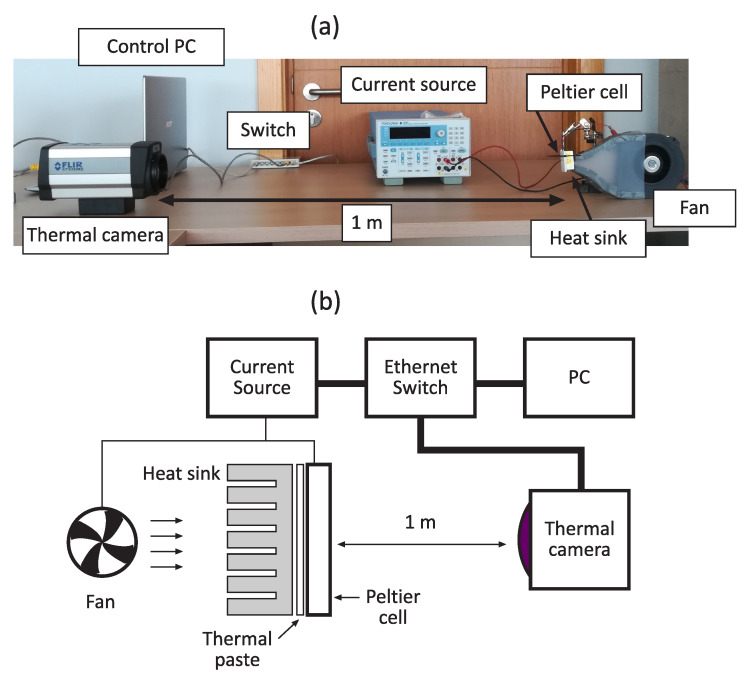
Photo of the experimental setup (**a**). Schematic of the experimental setup (**b**).

The experimental set-up (Figure 3) comprised a thermographic camera, a 8 cm${}^{2}$ Peltier cell, an Ethernet-controlled current source, an Ethernet switch, and a PC. Peltier effect is reversible depending on the polarization current’s direction. Therefore, any side can behave as a cooler or heater. However, for these experiments only the cold side has been considered for transmission purposes since a heat sink was coupled to the Peltier cell’s hot side using thermal paste. The use of the heat sink improves dissipation and therefore the maximum achievable temperature difference between layers, although it introduces thermal inertia, which would limit the maximum bandwidth. In addition, in order to further enhance dissipation (and hence the maximum achievable temperature gradient), forced air was introduced in a subset of experiments.

The Peltier cell’s cold side is affected by Peltier effect, Joule dissipation, and heat transfer from the hot side. Therefore, the better the hot side dissipation, the higher the maximum achievable driving current $I_{max}$ without significant effects on the cold side. The maximum allowed current $I_{max}$ was empirically obtained for both forced air and no-air-flow conditions, resulting in 3 A and 1 A, respectively.

Table 1 summarizes all the characteristics of the experimental set-up. The thermal camera’s capture rate was set to 12.4 fps, which was the allowed maximum.

For running the experiments, an automated script that governed both thermal camera and current source was executed. After the initialization of the current source and the thermal camera, all the start–stop combinations within the current sweep range (Table 1) were tested. Each iteration let the Peltier cell stabilize at the initial current $I_{0}$. Then, the current source was set to the final current value $I_{f}$ and 1000 frames were buffered using the thermographic camera for further processing.

The measured step response curves T(t) were fitted, and the system’s settling time $t_{s}$ was obtained as a performance metric. Regarding noise characterization ($\sigma_{N}^{2}$), the signal variance of the thermal images was estimated using the steady-state part of the curves. Using these metrics, an estimation of the Bit Error Rate (BER) for a given modulation/encoding could be provided.

Finally, the energy efficiency of this new paradigm (measured in bits per Joule) was analyzed and compared to the OCC’s. The comparison also considered other aspects such as the angular sensitivity and the impact of the receiver’s parameters. In order to carry out a fair comparison, the power consumption was compared assuming that both schemes presented the same BER for an On-Off Keying (OOK) encoding.

## 5. Results

**Figure 4:**
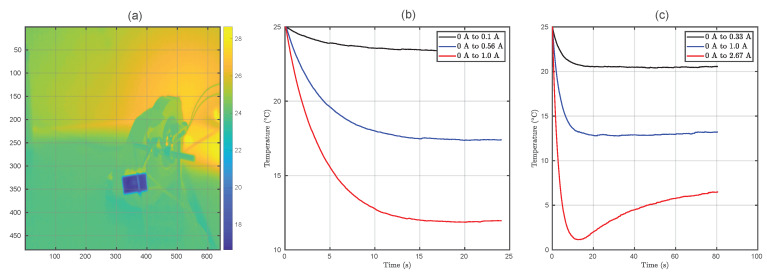
Example of captured thermographic image (**a**). For a driving current of 0.5 A without the fan activated, the cold side temperature reached 17 ${}^{{}^{\circ}}$C. Temperature step responses with passive cooling (**b**). Temperature step responses with active cooling using a fan (**c**).

Figure 4 shows an example of captured image in which the cold side of the Peltier cell can be observed. All the results shown in this section are obtained using these kind of captured images.

### 5.1. Curve Fitting

The first part of the analysis is based on obtaining a reasonable curve fitting of the temperature step response T(t). Figure 4 depicts the obtained step responses for both no-air-flow and forced-air boundary conditions. These curves were smoothed taking advantage of the spatial locality principle (temperature at close points over the Peltier cell will be the same) by averaging a window of 25 pixels.

The system’s step responses were fitted according to the curve presented in Equation (6), in which is easy to find that it defines a nonlinear system:

(6) $$T\left(t\right)=T_{0}+\Delta T\left(1-\left(1-\beta_{1}t^{\beta_{2}}\right)e^{-t/\tau }\right)$$

where T(t) is the temperature respect time, $T_{0}$ is the initial temperature, ΔT is the final temperature change induced by the current step change, τ is the time constant of the system which models heat propagation, $\beta_{1}$ is a term that models overshot, and $\beta_{2}$ describes the non-linearity of the system. ΔT depends on the driving current, which affects both Peltier effect and Joule dissipation. All the other parameters, which model the transient behavior of temperature, depend on both $I_{0}$ and $I_{f}$. A high goodness-of-fit metric was obtained for all the cases (the minimum coefficient of determination $R^{2}$ was 0.9414 and the average 0.988). These results suggest that the thermoelectric coefficients involved on the nonlinear equation which governs these systems cannot be assumed constant with temperature, at least for high currents.

Both $\beta_{1}$ and $\beta_{2}$ are coefficients involved into the size and shape of the overshot. It has been observed that $\beta_{2}$ is bound to the interval (0,1). Furthermore, for small currents, this exponent is close to 1, indicating a more linear behavior of the system. However, as the initial or final current increases, the overshot is emphasized and the exponent is reduced (more nonlinear behavior). Moreover, the presence of convection-aided cooling on the heat sink accentuates the non-linearities on the step response for a given driving current.

### 5.2. Bandwidth

**Figure 5:**
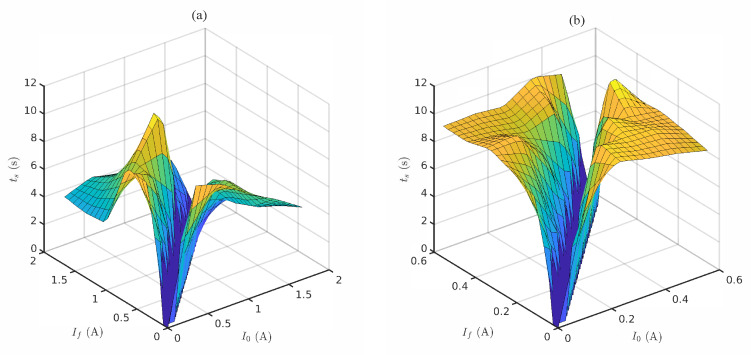
Settling times at 95% for a subset of parameters for both cooling boundary conditions. Active cooling (**a**) and passive cooling (**b**) boundary conditions were taken into account.

Despite the evident nonlinear behavior of the system, it would be possible to consider the system’s response as linear under a sufficiently slow pulsed-current regime. Nonetheless, frequency-domain analysis is not well suited due to the nonlinear behavior of the system. Therefore, time-domain analysis is preferred in this case. Figure 5 depicts the system’s settling time for different $I_{0}$ and $I_{f}$ values.

It can be observed that active cooling dramatically improves settling time. In addition, there is a significant difference between cooling ($I_{f}>I_{0}$) and heating times ($If<I_{0}$). Therefore, depending on the selected current values, the bit duration should be limited to the slowest one (Equation (7)).

(7) $$T_{b}=\max\left\{t_{s}\left(I_{0},I_{f}\right),t_{s}\left(I_{f},I_{0}\right)\right\}$$

**Figure 6:**
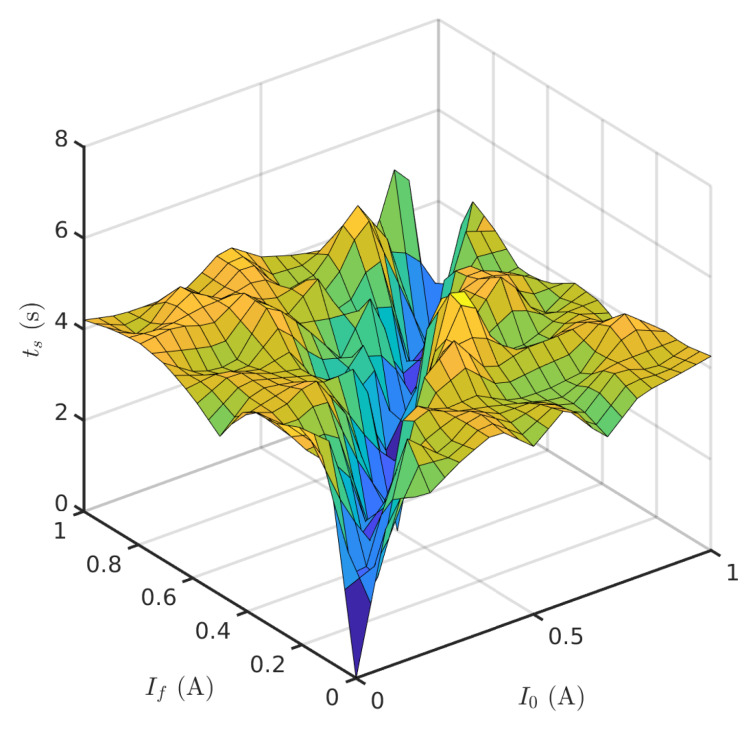
Settling times at 95% for a subset of parameters for the small excitation regime with passive cooling.

It has been observed that bandwidth depends directly on the average temperature (defined by $I_{0}$ and $I_{f}$), which can be understood as a polarization point. Nonetheless, for small-signal excitation (below 100 mA), the Peltier cell behaved as a first-order system and the measured settling time did not depend on the driving current, resulting in an approximate bandwidth $B_{TCC}$ of 0.12 Hz (±0.015 Hz). Figure 6 illustrates the settling time for small driving currents and Equation (8) shows the mathematical relationship between settling time at 95% and bandwidth in first order systems. As OOK is being considered as a first approximation, the achievable data rate would be equal to the system’s bandwidth.

(8) $$B_{TCC}\approx \frac{3}{2\pi t_{s}}$$

### 5.3. Bit Error Rate

Regarding receiver noise, the variance of the captured signals was calculated at their steady-state regime using 50 samples. It was observed that for all cases, $\sigma_{N}^{2}$ was approximately 2.2 · 10${}^{-3}$ K${}^{2}$. An equality test for variances (Snedecor–Fisher test) was carried out by pairs, resulting in no significant differences (maximum *p*-value 0.0289). This implies that noise depends only on the receiver’s characteristics, unlike OCC, in which noise depends also on the incident optical power. Nevertheless, as it was performed on T(t)’s curve fitting, it is possible to reduce noise by averaging a window thanks to the use of image-forming optics, which results on the spatial confinement of light sources within the thermal image. It must be taken into account that this noise reduction technique is limited by the projected size of the Peltier’s cold side on the microbolometer sensor.

**Figure 7:**
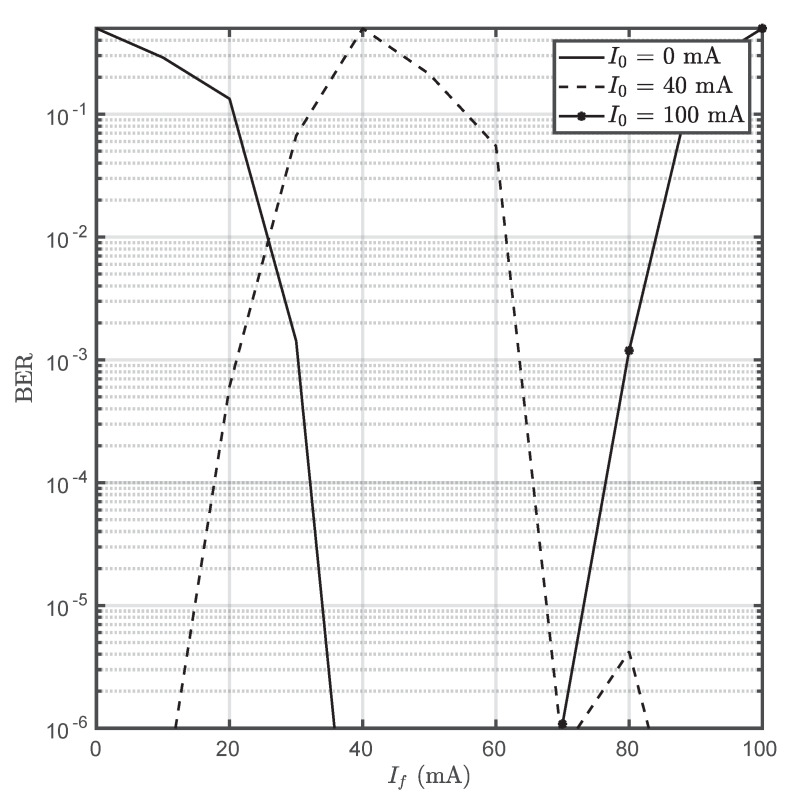
Bit Error Rate (BER) for different $I_{0}$ and $I_{f}$ values. Both currents (and therefore resulting temperatures) can be considered as logical 0 or logical 1 without loss of generality.

In order to illustrate the communications feasibility of TCC, BER curves have been estimated using different parameters for an OOK scheme. Figure 7 shows that small differences on the driving current ($I_{f}-I_{0}$) generate enough temperature variation on the Peltier cell to induce a very small BER on the receiver (thanks to the low noise of the thermal camera). This leads to the straightforward conclusion that active cooling is not necessary and would dramatically affect energy consumption. Due to the limited data rate of the system and the small expected BER, which would had needed from very long times, a mathematical estimation using the available data was preferred rather than an experimental evaluation. Equation (9) provides the mathematical estimation of the BER [28].

(9) $$BER=\frac{1}{2}\mathrm{erfc}\left(\frac{1}{\sqrt{2}}\frac{|T_{0}-T_{f}|}{2\sigma_{N}}\right)$$

where erfc(·) is the complementary error function, and $T_{0}$ and $T_{f}$ are the temperatures associated to the logical 0 and 1.

**Figure 8:**
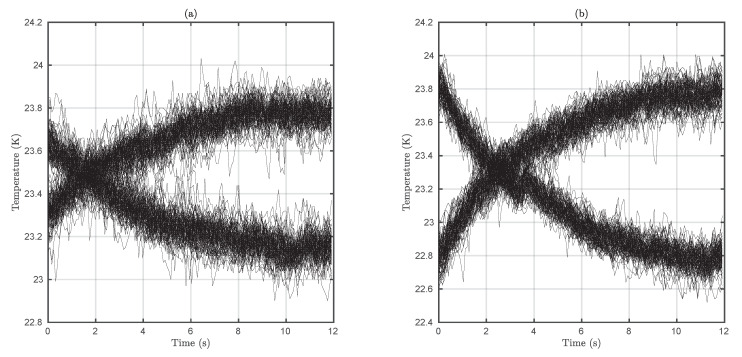
Eye patterns for different $I_{f}$ values with $I_{0}$ equal to zero (most energy efficient configuration). (**a**) $I_{f}$ set to 30 mA. (**b**) $I_{f}$ set to 60 mA.

Finally, Figure 8 depicts two exemplary experimental eye patterns which show the time histogram of the system in presence of the thermographic camera’s inherent noise. It can be observed that the eye opens as the current increases. Furthermore, the slope is insensitive to the current (at least in small signal regime) and jitter has a very reduced magnitude.

### 5.4. Energy Efficiency

**Figure 9:**
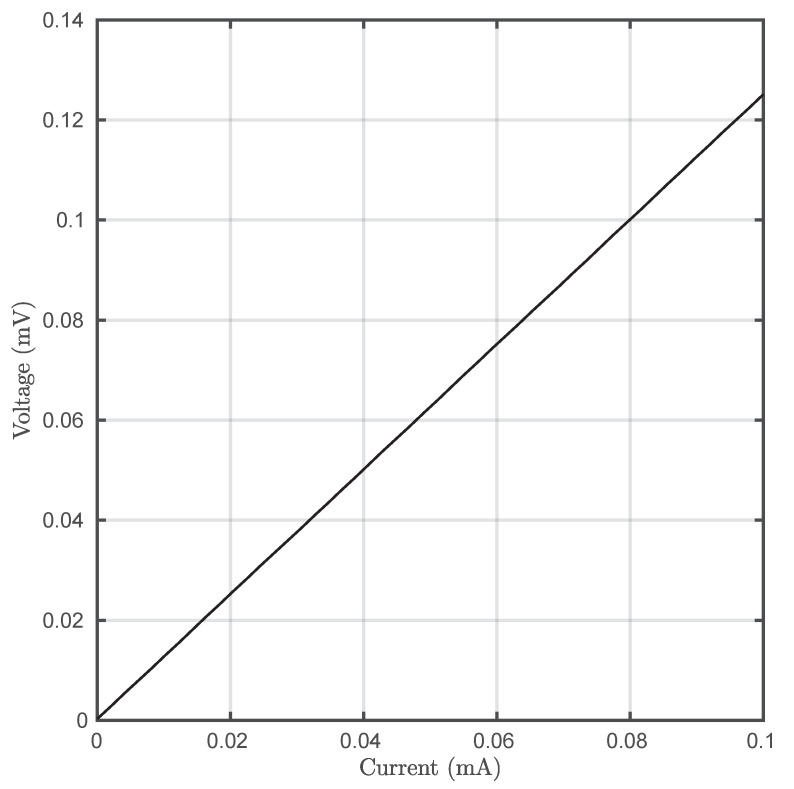
Voltage–current curve of the used Peltier cell.

**Figure 10:**
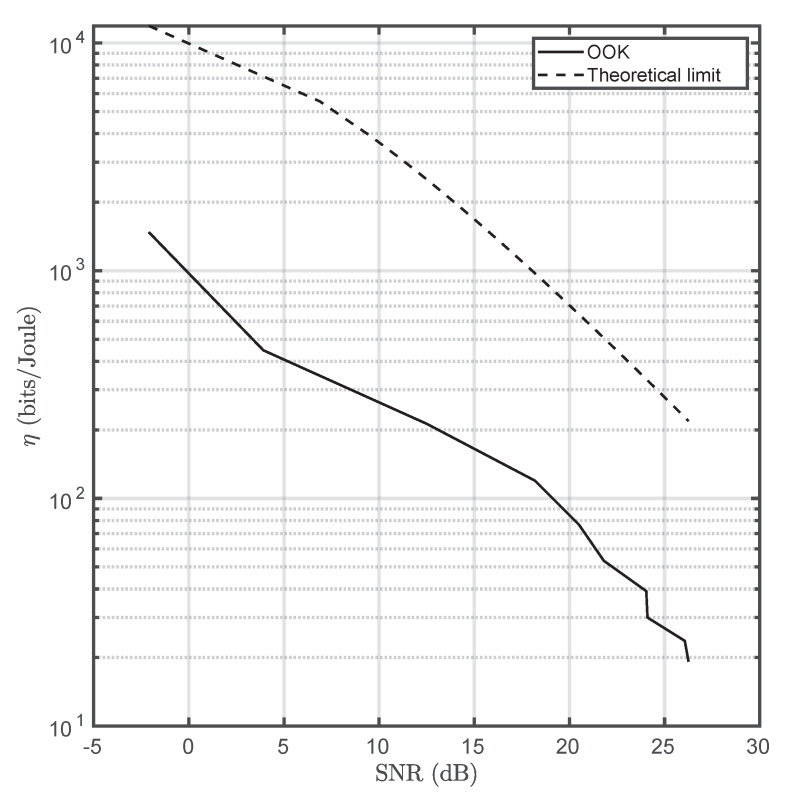
Energy efficiency for different values of SNR versus the theoretical limit imposed by Shannon–Hartley’s theorem.

Energy efficiency is a communications aspect that is generally underestimated, and will be denoted as η hereinafter. Nevertheless, for energy-scarce scenarios, such as wireless sensor network deployments, this aspect is capital and sometimes defines the network’s lifespan. In this work, this parameter has been estimated using the number of correctly transmitted bits per Joule (Equation (10)) [29]. In order to properly calculate energy consumption, voltage was recorded using the programmable current source (Figure 9). Figure 10 depicts η as a function of the induced *SNR*.

(10) $$\eta =\xi \frac{B}{E\left[P\right]}\left(1-BER(SNR)\right)$$

where ξ is the spectral efficiency of the used modulation (1 for OOK), *B* is bandwidth, and E[P] is the average power consumption (Equation (11)).

(11) $$E\left[P\right]=\frac{I_{0}V_{0}+I_{f}V_{f}}{2}$$

Only the most energy-efficient combinations of $I_{0}$ and $I_{f}$ was considered. These combinations are associated to the use of $I_{0}=0$ mA for obvious reasons regarding power consumption.

## 6. Comparison with OCC

TCC has been proposed as a suitable low-speed communications technology. Nonetheless, in order to assess the actual feasibility of this novel paradigm, it must be fairly compared to a similar well-studied alternative such as OCC. In this section, TCC is being compared to OCC assuming that the reception is carried out using Global Shutter (GS) instead of RS as its performance is highly affected by both emitter size and link range. Therefore, OCC data rate ($B_{OCC}$) will be half the frame rate (fps) in the best case. Moreover, a Single Input Single Output (SISO) scheme has been considered in this work without loss of generality.

Assuming that in both cases the used cameras present the same optical system, pixel pitch, and resolution, the comparison would just focus on the differences between the emission and reception efficiencies. This is summarized in the following inequality,

(12) $$\frac{B_{TCC}}{E\left[P_{TCC}^{\left(elec\right)}\left(SNR\right)\right]}>\frac{B_{OCC}}{E\left[P_{OCC}^{\left(elec\right)}\left(SNR\right)\right]}$$

where $P_{OCC}^{\left(elec\right)}$ and $P_{TCC}^{\left(elec\right)}$ are the electrical power consumption of OCC and TCC, respectively. To induce a given SNR on the receiver, a certain amount of power is needed to be consumed on the emission side. As OOK is being considered for simplicity, SNR can be easily related to emitted optical power (Equations (13) and (14)),

(13) $$\begin{array}{cc} SNR_{OCC} & =\frac{1}{2}{\left(\frac{P_{OCC}\cdot H_{OCC}\cdot T_{exp}}{\sigma_{OCC}}\right)}^{2}\\ \; & \end{array}$$

(14) $$\begin{array}{cc} SNR_{TCC} & =\frac{1}{2}{\left(\frac{P_{TCC}\cdot H_{TCC}}{\sigma_{TCC}}\right)}^{2} \end{array}$$

where $P_{OCC}$ and $P_{TCC}$ are the radiated optical powers in both schemes, whereas $H_{TCC}$ and $H_{OCC}$ are channel gains. These coefficients have been assumed constant. Regarding noise, $\sigma_{OCC}$ and $\sigma_{TCC}$ are expressed in electrons (e${}^{-}$) and Kelvin, respectively. $T_{exp}$ is the frame exposure time of the CMOS camera. Furthermore, the radiated optical powers are directly related to the driving currents via the conversion efficiencies $\kappa_{LED}$ and $\kappa_{Peltier}$, which act as current-dependent coefficients. In the case of TCC, the product $H_{TCC}\cdot \kappa_{Peltier}$ was observed to be approximately constant, at least under small-signal regime. Including the conversion efficiencies in Equations (13) and (14) it yields Equations (15) and (16), respectively.

(15) $$\begin{array}{cc} SNR_{OCC} & =\frac{1}{2}{\left(\frac{I_{OCC}\cdot \kappa_{LED}\cdot H_{OCC}\cdot T_{exp}}{\sigma_{OCC}}\right)}^{2}\\ \; & \end{array}$$

(16) $$\begin{array}{cc} SNR_{TCC} & =\frac{1}{2}{\left(\frac{I_{TCC}\cdot \kappa_{Peltier}\cdot H_{TCC}}{\sigma_{TCC}}\right)}^{2} \end{array}$$

Power consumption to induce a given SNR, $P^{\left(elec\right)}\left(SNR\right)$, can be easily obtained in both schemes by expressing the driving current in terms of the other parameters. Equations (17) and (18) show the associated mathematical description.

(17) $$\begin{array}{cc} P_{OCC}^{\left(elec\right)}\left(SNR\right) & =2SNR\cdot R_{OCC}{\left(\frac{\sigma_{OCC}}{H_{OCC}\cdot \kappa_{LED}\cdot T_{exp}}\right)}^{2}\\ \; & \end{array}$$

(18) $$\begin{array}{cc} P_{TCC}^{\left(elec\right)}\left(SNR\right) & =2SNR\cdot R_{TCC}{\left(\frac{\sigma_{TCC}}{H_{TCC}\cdot \kappa_{Peltier}}\right)}^{2} \end{array}$$

Including Equations (17) and (18) into (12), it yields the following final expression.

(19) $$\frac{B_{TCC}}{R_{TCC}}{\left(\frac{H_{TCC}\;\kappa_{Peltier}}{\sigma_{TCC}}\right)}^{2}>\frac{B_{OCC}}{R_{OCC}}{\left(\frac{H_{OCC}\;\kappa_{LED}\;T_{exp}}{\sigma_{OCC}}\right)}^{2}$$

$R_{TCC}$ and $R_{OCC}$ are the equivalent resistances of both types of emitter at a given current. In the case of TCC, Figure 9 demonstrated that Peltier cells present Ohmic behavior at least for small signal regime ($R_{TCC}$ approximately constant). However, as LEDs are used in OCC and they present strong nonlinear behavior with current, $R_{OCC}$ would tend to decrease.

In order to carry out the comparison, $H_{TCC}$, $\kappa_{TCC}$, $\sigma_{TCC}$, and $R_{TCC}$ were obtained from the experimental results of this work. $\sigma_{OCC}$ has been assumed to be comparable to a high-end camera’s noise performance under a low exposure time regime, whereas $R_{OCC}$ and $\kappa_{LED}$ have been obtained from a commercial LED device. For comparison purposes, the receiver’s quantum efficiency (QE) has been set to 0.5 (corresponding to a 660 nm emission). Finally, $H_{OCC}$ has been estimated using Equation (20) [27], which takes into account the optical camera’s angular resolution $\varphi_{xy}$ (Equation (21)).

(20) $$\begin{array}{cc} H_{OCC} & =\frac{1}{2\pi }\frac{A_{lens}}{A_{tx}\;\varphi_{xy}}A_{pixel}\frac{QE}{E_{ph}}\\ \; & \end{array}$$

(21) $$\begin{array}{cc} \varphi_{xy} & =\frac{1}{4}\frac{N_{x}\;N_{y}}{FOV_{x}\;FOV_{y}} \end{array}$$

**Figure 11:**
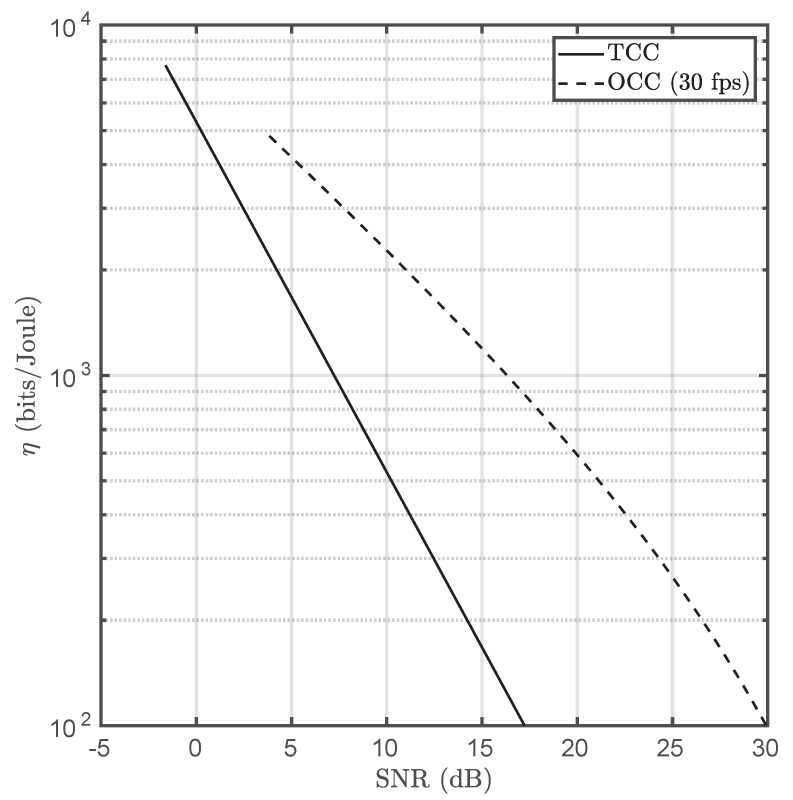
Energy efficiency comparison between TCC and OCC for a frame rate of 30 fps.

$H_{OCC}$ has units of electrons per Joule, and $E_{ph}$ is the arriving photon energy. It has been assumed that the emitting optical surface presents the typical Lambertian profile. Moreover, the system has no misalignment and is perfectly focused. $A_{lens}$, $A_{tx}$, and $A_{pixel}$ are the main lens, optical emitter, and pixel areas, respectively. $N_{x}$ and $N_{y}$ are the image sensor’s horizontal and vertical resolutions, respectively. $FOV_{x}$ and $FOV_{y}$ are the horizontal and vertical fields of view, respectively. The channel gain expression is valid for emitters fully projecting at least on 1 pixel. Table 2 summarizes all the used parameters and Figure 11 illustrates the energy efficiencies of TCC and OCC for a frame rate of 60 fps.

The power consumption of the OCC system has been scaled up by a factor of 40, resulting from the ratio between the Peltier cell’s area and the LED cross section. Under this assumption, the OCC transmitter would comprise an LED matrix. This has been included into the comparison in order to ensure that both systems present the same working range, which is limited by the transmitter area in camera-based communications.

OCC’s energy efficiency is greater than TCC’s. However, it must be highlighted that Peltier cell design is not optimized for communications but only for thermal applications. In addition, thermoelectric coolers consume more power depending on their area, and their bandwidth is closely related to their thickness. Despite the current energy-efficiency gap, Peltier-based TCC emitters present hemispherical radiance as they are based on blackbody radiation, which turns them insensitive to angular deviations. On the other hand, OCC is generally affected by misalignment due to the directivity of LED emitters. Furthermore, OCC’s performance is highly dependent on the camera’s parameters such as exposure time and analog gain. This does not occur in TCC, at least for indoor applications in which atmospheric absorption is negligible. Finally, notwithstanding the claim about the dual use of cameras is OCC (e.g., simultaneous surveillance and data acquisition), this does not happen in practical implementations as very low exposure times are needed to properly detect and decode the light sources within the scene (background mitigation). In contrast, TCC could be a priori a strong candidate for providing both applications simultaneously, but more research is needed in this aspect.

## 7. Conclusions

In this work, the use of thermographic cameras as communications receivers has been proposed and analyzed. It has been demonstrated that microbolometer-based thermal cameras are suitable devices for communications. In addition, Peltier cells have been also proposed as current-driven optical emitters.

Peltier cells present nonlinear behavior respect to the driving current. Nevertheless, it has been observed that for step-like excitation, the current-temperature response exhibits an overshot that emphasizes with the presence of a forced-air flow on the heat sink. However, the extra power consumption of this active cooling strategy turns it unfeasible from an energy-efficiency viewpoint. Regarding bandwidth, it has been shown that it depends on the driving current in the large signal regime (more than 100 mA) and on the heat dissipation capacity of the Peltier cell’s hot side. For small currents (below 100 mA), it was observed that bandwidth remained stable around 0.12 Hz for the 8 cm${}^{2}$ used Peltier cell. This reduced bandwidth performance depends on several factors, and is closely related to the lack of optimization of these devices as communication endpoints. More research is needed in this regard, and a communications-optimized Peltier cell may be produced optimizing its thickness, number of semiconductor pellets, and its enclosing material (some thermal inertia could be alleviated by removing the typical outside ceramic layers).

The temperature noise on the thermographic camera has been shown to be stable respect to the emitter’s temperature, unlike it occurs on optical cameras, suggesting that it is a parameter which depends only on the receiver’s characteristics. Furthermore, small driving currents (35 mA) produced enough SNR on the receiver to induce a BER below 10${}^{-6}$. Nonetheless, the consumed current could be dramatically reduced as it occurs regarding bandwidth with some optimization efforts. It must be highlighted that current consumption is intimately related to the Peltier cell’s area, and a commercial thermoelectric cooler has been used as device under test in this work.

The energy efficiency of this technology has been also analyzed. As bandwidth is not related to the driving current in the small signal regime (the most energy-efficient), the energy efficiency does not depend on this parameter. However, 530 bits per Joule can be achieved inducing an SNR of 10 dB on the thermographic camera. This result is conditioned to the Peltier cell’s size, as, due to the fabrication process, bigger cells present higher power consumption as it is aforementioned. However, there is a trade-off between power consumption and maximum communication range.

TCC has been shown to be less energy-efficient than OCC by a factor between 4 and 6, depending on the SNR. However, as it was already commented, TCC emitters are not optimized for data transmission. In spite of this difference, TCC in insensitive to misalignment since it is based on the constant radiance of black bodies. Furthermore, OCC receivers are not capable of operating simultaneously as sensor data acquisition endpoints and general purpose cameras due to the sensitivity of the reception performance with respect to exposure time and analog gain. This does not happen in TCC, removing the necessity of determining the optimal exposure-gain combination to maximize SNR (or even allow reception due to saturation).

It has been demonstrated that this technology can be used not only for traditional thermal applications, but also for communications. The new explored capabilities of both thermal imaging and Peltier cells in this work may be of great utility in the near future, as 2020’s health emergency will make the use of thermographic cameras ubiquitous.

Moreover, the current characterization has considered only open-loop control on the transmitter, which leads to very low rate capabilities. Nevertheless, observing the results from Figure 4 and Figure 5, it can be inferred that the use of nonlinear control could significantly improve settling time, and hence throughput. Furthermore, this technology could be combined with spatially-modulated arrangements using small-size Peltier cells, further enhancing the capabilities of this novel application of thermography.

## Figures and Tables

**Table 1:** Characteristics of the experimental setup.

Parameter	Value
Camera model	FLIR A645 LWIR
Camera frame rate	12.4 fps
Camera focus distance	1 m
Camera resolution	640 × 480 pixels
Temperature resolution	50 mK
Peltier cell area	4 × 2 cm${}^{2}$
Peltier cell depth	4 mm
Peltier material	Bi${}_{2}$Te${}_{3}$
Heat sink area	8 cm${}^{2}$
Heat sink material	6063 aluminium alloy
Current source model	Yokogawa GS820
$I_{max}$	1.0 A (no air flow)
	3.0 A (forced air)
Current sweep points	100

**Table 2:** TCC and OCC parameters used to carry out the energy efficiency comparison.

Parameter	Value
$H_{TCC}\cdot \kappa_{Peltier}$	16.6 K/A
$R_{TCC}$	1.256 Ω
$\sigma_{TCC}$	50 mK
$H_{OCC}$	1.165 · 10${}^{10}$ e${}^{-}$/J
$\kappa_{LED}$	≈1 mW/mA
$\sigma_{OCC}$	[30]
$R_{OCC}$	[31]
$A_{tx}$	19.63 · 10${}^{-6}$ m${}^{2}$ (5 mm LED)
$A_{lens}$	1.347 · 10${}^{-4}$ m${}^{2}$
$A_{pixel}$	1.25 · 10${}^{-12}$ m${}^{2}$ (1.12 μm pitch)
$T_{exp}$	1/10,000 s
QE	0.5
$E_{ph}$	1.883 eV
$\left(N_{x},N_{y}\right)$	(640, 480)
$\left(FOV_{x},FOV_{y}\right)$	(45${}^{{}^{\circ}}$, 34${}^{{}^{\circ}}$)
Dark current	14.4 e${}^{-}$/s
Readout noise	32 e${}^{-}$
